# Alpha-1 Antitrypsin and Hepatocellular Carcinoma in Liver Cirrhosis: *SERPINA1* MZ or MS Genotype Carriage Decreases the Risk

**DOI:** 10.3390/ijms221910560

**Published:** 2021-09-29

**Authors:** Zuzana Rabekova, Sona Frankova, Milan Jirsa, Magdalena Neroldova, Mariia Lunova, Ondrej Fabian, Martin Kveton, David Varys, Klara Chmelova, Vera Adamkova, Jaroslav A. Hubacek, Julius Spicak, Dusan Merta, Jan Sperl

**Affiliations:** 1 Department of Hepatogastroenterology, Institute for Clinical and Experimental Medicine, 140 21 Prague, Czech Republic; zuzana.rabekova@ikem.cz (Z.R.); david.varys@ikem.cz (D.V.); klara.chmelova@ikem.cz (K.C.); julius.spicak@ikem.cz (J.S.); 2 First Faculty of Medicine, Charles University, 120 00 Prague, Czech Republic; magdalena.neroldova@ikem.cz; 3 Laboratory of Experimental Hepatology, Experimental Medicine Centre, Institute for Clinical and Experimental Medicine, 140 21 Prague, Czech Republic; milan.jirsa@ikem.cz (M.J.); mariia.lunova@ikem.cz (M.L.); 4 Institute of Medical Biochemistry and Laboratory Diagnostics, First Faculty of Medicine, Charles University, 120 00 Prague, Czech Republic; 5 Clinical and Transplant Pathology Department, Institute for Clinical and Experimental Medicine, 140 21 Prague, Czech Republic; ondrej.fabian@ikem.cz (O.F.); martin.kveton@ikem.cz (M.K.); 6 Preventive Cardiology Centre, Institute for Clinical and Experimental Medicine, 140 21 Prague, Czech Republic; vera.adamkova@ikem.cz; 7 Atherosclerosis Research Laboratory, Institute for Clinical and Experimental Medicine, 140 21 Prague, Czech Republic; jaroslav.hubacek@ikem.cz; 8 Anaesthesiology and Resuscitation Department, Institute for Clinical and Experimental Medicine, 140 21 Prague, Czech Republic; dusan.merta@gmail.com; 9 Department of Internal Medicine, 1st Faculty of Medicine, Charles University and Military University Hospital, 160 00 Prague, Czech Republic

**Keywords:** alpha-1-antitrypsin, *SERPINA1* gene, cirrhosis, hepatocellular carcinoma, Z allele, S allele

## Abstract

Heterozygotes for Z or S alleles of alpha-1-antrypsin (AAT) have low serum AAT levels. Our aim was to compare the risk of hepatocellular carcinoma (HCC) in patients with liver cirrhosis carrying the *SERPINA1* MM, MZ and MS genotypes. The study groups consisted of 1119 patients with liver cirrhosis of various aetiologies, and 3240 healthy individuals served as population controls. The MZ genotype was significantly more frequent in the study group (55/1119 vs. 87/3240, *p* < 0.0001). The MS genotype frequency was comparable in controls (32/119 vs. 101/3240, *p* = 0.84). MZ and MS heterozygotes had lower serum AAT level than MM homozygotes (medians: 0.90 g/L; 1.40 g/L and 1.67 g/L; *p* < 0.001 for both). There were significantly fewer patients with HCC in the cirrhosis group among MZ and MS heterozygotes than in MM homozygotes (5/55 and 1/32 respectively, vs. 243/1022, *p* < 0.01 for both). The risk of HCC was lower in MZ and MS heterozygotes than in MM homozygotes (OR 0.3202; 95% CI 0.1361–0.7719 and OR 0.1522; 95% CI 0.02941–0.7882, respectively). Multivariate analysis of HCC risk factors identified MZ or MS genotype carriage as a protective factor, whereas age, male sex, BMI and viral aetiology of cirrhosis increased HCC risk.

## 1. Introduction

Alpha-1 antitrypsin (AAT), encoded by *SERPINA1* gene, is an acute phase reactant and major plasma serine protease inhibitor (Pi) [1]. The principal role of AAT is to protect lung tissue against damage by neutrophil elastase. *SERPINA1,* located on 14q32.13, spans ~14 kb. Plasma AAT is mainly contributed by the liver, and some AAT is also synthetized in intestinal and pulmonary alveolar cells, kidney, neutrophils, macrophages and, to a lesser extent, in numerous other tissues. Tissue-specific expression of AAT is attributed to complex organisation and splicing of the 5′-UTR. Eleven splicing variants, all coding the same AAT protein, differ in number (1 to 3) and range of the non-coding exons. The gene has two transcription start sites and its expression is driven by two different promoters—upper macrophage-specific and lower hepatocyte-specific. Macrophage-specific promoter is silent in hepatocytes. Tissue specific expression of AAT is attributed to different patterns of splice variants with different translational activity [2,3]. The contribution of promoter hypermethylation in peripheral blood monocytes has also been documented [4].

The wild-type AAT allele named M represents any of the five polymorphic alleles M1A, M1V, and M2–M4 with identical AAT protein mobility on isoelectric focusation. The MM genotype is present in about 88% of the general population. Variant pathogenic alleles are divided in three subtypes. The first type is the storing alleles represented by the second most frequent variant allele Z (*p*.Glu366Lys, rs28929474 c.1096G>A) and by two rare alleles M_malton_ (*p*.Phe76del, rs775982338 c.226_228delTTC) and S_iiyama_ (*p*.Ser77Phe, rs55819880 c.230C>T) [5,6]. Proteins resulting from the storing alleles undergo partial intracellular degradation (~70%), secretion (~15%) and polymer formation (~15%) [7,8,9]. Only a small fraction of polymers are degraded or secreted out of the cell. The rest persist in the endoplasmic reticulum (ER) and the deposits are detectable as diastase-resistant inclusions, positive in periodic acid-Schiff staining (PASD positive) [10]. Polymer deposition in ER causes impairment of ER function, leading to liver steatosis and fibrosis [11]. The second type is null (QO) alleles, characterized by absence of protein expression or by synthesis of a non-polymerizing truncated protein. The S allele (*p*.Glu288Val, rs17580 c.863A>T), being the most frequent variant allele in Caucasians, represents the third type, which is characterised by the synthesis of a dysfunctional protein undergoing complete intracellular degradation. The minor allele frequencies of alleles S and Z in Caucasians are 3.7% and 1.6%; according to the dbSNP Allele Frequency Aggregator, a total of additional 132 rare genetic variants within the coding sequence, defined as 120 low-frequency mutations (<1% in the general population), have also been identified [12,13,14].

Whereas S allele or null alleles are not associated with liver disease, the storing variants are; the association of lung emphysema and liver cirrhosis in ZZ homozygotes is well documented [15]. A cross-sectional biopsy study and a large European study based on non-invasive assessment of liver fibrosis in ZZ homozygotes brought similar results—clinically significant liver fibrosis in 35% and 20–36%, respectively [16,17]. The absolute risk of liver cirrhosis in MZ heterozygotes has not been established so far; however, it increased the risk of liver cirrhosis in studies on patients with chronic liver disease of various aetiologies [18]. The impact of the MZ genotype on the risk of liver cirrhosis was apparent in patients with alcoholic liver disease and non-alcoholic steatohepatitis, but it was not confirmed in patients with cholestatic liver diseases and liver cirrhosis owing to viral hepatitis [19,20]. It seems that the MZ genotype increases the risk of liver cirrhosis in chronic liver diseases in which impaired lipid trafficking through the ER plays a substantial role. AAT also cleaves various proteins, including the innate immunity proteins.

The interaction between AAT and tumour necrosis factor alpha (TNF-alpha) was the subject of many studies, especially in patients with the AATD-associated lung disease. The interaction between AAT and TNF-alpha is complex and involves four main mechanisms whereby AAT can modulate TNF-alpha signalling [21]. Firstly, AAT appears to modulate TNF-alpha gene self-expression in both endothelial cells and neutrophils [22]. Secondly, in AATD, the misfolded Z AAT was shown to accumulate in the ER of neutrophils and ultimately contribute to ER stress, leading to the expression of pro-apoptotic cytokines, including TNF-alpha. Thirdly, AAT can regulate the activity of ADAM metallopeptidase domain 17 (ADAM-17), a matrix metalloprotease also known as TNF-alpha, converting enzyme (TACE) in endothelial cells and neutrophils, thereby reducing the amount of transmembrane TNF-alpha (mTNF-alpha) converted to soluble TNF-alpha (sTNF-alpha) on the cell surface [23]. Fourthly, AAT can bind TNF-alpha cognate receptors TNFR1 and TNFR2, preventing interaction and subsequent downstream signalling, thereby modulating cell priming and degranulation in neutrophils [24]. AAT showed also a significant anti-apoptotic function in lung endothelial cells by the direct inhibition of caspases-3, -6 and -7 [25,26,27]. Obviously, a loss or decreased level of AAT can result in a pro-inflammatory and pro-apoptotic status [28,29]. MZ genotype carriage is also associated with immune disorders, such as ANCA-associated vasculitis [30]. ANCA-associated vasculitis occurs also with other variant alleles, such as allele S [31]. This fact indicates an association of the immune-mediated disease with decreased AAT level and/or function.

Some authors consider AAT deficiency to be a risk factor of hepatocellular carcinoma (HCC). This opinion is supported by anecdotal papers and by mouse studies [32,33]. Support for the opposite opinion comes from an American retrospective study that included only patients with advanced liver cirrhosis [34]. The incidence of HCC during a median observation period of 3.4 years was lower (8.5%) in the AAT deficiency group (47 carriers of the Z allele) than in 628 patients with the MM genotype (31%). Encouraged by this study, we postulated a hypothesis that pro-inflammatory and pro-apoptotic immune mechanisms in subjects with low AAT level could protect against HCC in liver cirrhosis and assessed the risk of HCC in MZ and MS genotype carriers in a large group of patients with advanced liver cirrhosis of various aetiologies.

## 2. Results

### 2.1. Genotype Frequency in Cases and Controls

The frequencies of the particular genotypes are presented in Table 1. The frequency of the *SERPINA1* MZ genotype was significantly higher (*p* < 0.0001) in the cirrhosis group (55 of 1119; 4.92%) than in the controls (87 of 3240; 2.69%). The carriage of the *SERPINA1* MZ genotype increased the risk of liver cirrhosis (OR 1.986; 95% CI 1.413–2.806). The frequency of the *SERPINA1* MS genotype in the cirrhosis group (32 of 1119; 2.86%) and in the control group (101 of 3240; 3.12%) did not differ significantly, and the carriage of the *SERPINA1* MS genotype had no impact on the risk of liver cirrhosis.

When evaluating the genotype frequency in the subgroups of cases according to the liver cirrhosis aetiology, the frequency of the *SERPINA1* MZ genotype carriers was significantly higher in the subgroups of cases with ALD and NASH cirrhosis (24 of 360; 6.67% and 16 of 125; 12.8%, respectively, *p* < 0.001 for both) than in the controls (87 of 3,240; 2.69%). The frequency of the *SERPINA1* MZ genotype did not differ when comparing the pooled subgroups VIR, AIH-CHOL and MET with controls (15 of 569; 2.64% vs. 87 of 3240; 2.69%; N.S.). *SERPINA1* MZ genotype carriage increased the risk of liver cirrhosis only in patients with ALD (OR 2.745; 95% CI 1.744–4.325) and NASH (OR 4.729; 95% CI 2.733–8.298), as shown in Table 2. The frequency of the *SERPINA1* MS genotype differed neither in the ALD and NASH subgroup nor in the pooled other subgroup of cases from the frequency in the controls (9 of 327; 2.75%; 4 of 125; 3.2%; 19 of 569; 3.34% vs. 101 of 3240; 3.12%; N.S. for all). Carriage of the *SERPINA1* MS genotype did not increase the risk of liver cirrhosis in any of the subgroups of the cirrhotic patients.

### 2.2. Patients’ Demographic and Laboratory Data According to the SERPINA1 Genotype

**Figure 1:**
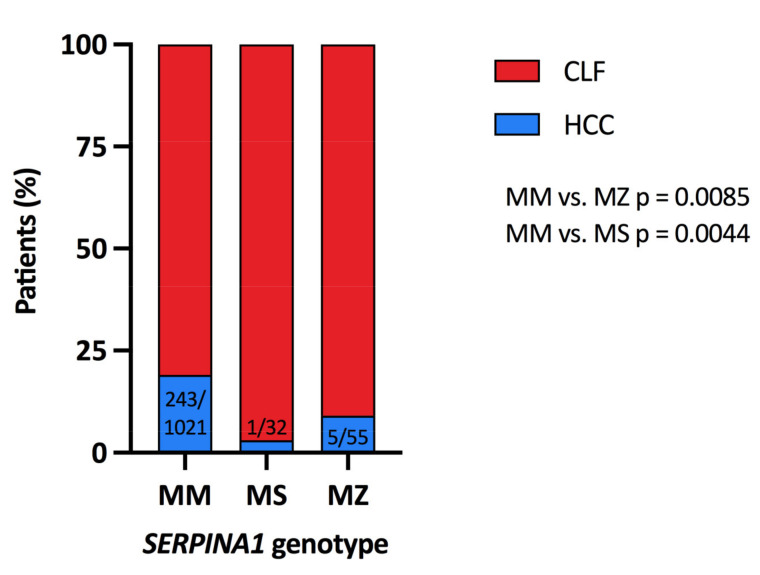
HCC in patients with liver cirrhosis according to the *SERPINA1* genotype. HCC, hepatocellular carcinoma; CLF, chronic liver failure.

As the study intended to evaluate the risk of liver cirrhosis and HCC associated with the *SERPINA1* MZ or MS genotype, only these genotypes were included in further analyses, and both heterozygotes were compared with the wild-type MM homozygotes. There were 249 patients with HCC (after exclusion of 2 cases with ZZ genotype) and 859 cases with liver cirrhosis without HCC. The patients with HCC were significantly older (median 62.6 years, range 26–75, vs. 53.6 years, range 18–74; *p* < 0.0001). There were significantly more males (176/249, 70.7%) among the patients with HCC than in the patients without HCC (472/859, 54.9%), *p* < 0.0001. Table 3 shows that the distribution of cases among aetiology subgroups in *SERPINA1* MS heterozygotes did not differ from the distribution in MM homozygotes. The MZ heterozygotes subgroup included significantly more patients with ALD and NASH cirrhosis (*p* = 0.002). HCC occurrence was significantly higher among the MM homozygotes (243 of 1021; 23.8%) than among the *SERPINA1* MZ (5 of 55; 9.1%) and MS heterozygotes (1 of 32; 0.4%). The carriage of both heterozygous genotypes (MZ or MS) seems to be protective against HCC development in the cirrhotic liver (OR 0.3202; 95% CI 0.1361–0.7719, *p* = 0.0085 and OR 0.1522; 95% CI 0.02941–0.7882, *p* = 0.0044; respectively), as depicted in Figure 1.

Further patients’ characteristics and laboratory data are presented in Table 4. Consistent with the fact that MZ genotype carriage represents a risk factor of liver cirrhosis, the MZ heterozygotes did not differ from MM homozygotes in age but they significantly differed in all the parameters determining liver dysfunction (MELD score, albumin and bilirubin level, prothrombin time, and creatinine). In accordance with the fact that the MZ heterozygotes group was enriched by cases with NASH cirrhosis, they had significantly higher BMI. Interestingly, the MS heterozygotes were younger than MM homozygotes and presented also with significantly worse parameters of liver dysfunction in comparison with MM homozygotes (MELD score, bilirubin, prothrombin time, creatinine) but they did not differ in serum albumin level.

**Figure 2:**
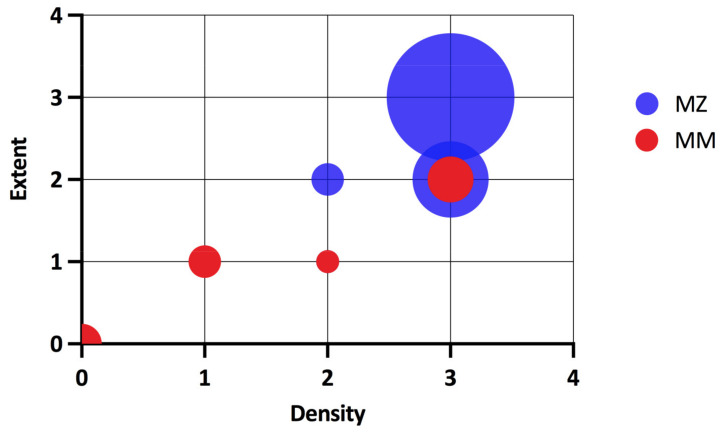
Extent and density of intrahepatic PASD positive aggregates, according to *SERPINA1* genotype. PASD, periodic acid–Schiff diastase.

In agreement with the finding that the variant Z protein forms polymer deposits in hepatocytes, the PASD positive aggregates were found significantly more often in the explanted liver of MZ heterozygotes than in those of MM heterozygotes (52 of 55; 94.5% vs. 18 of 953; 1.89%; *p* < 0.0001). The presence of PASD positive aggregates in MS heterozygotes did not differ from that in MM homozygotes (1 of 29; 3.45% vs. 18 of 953; 1.89%; N.S.). The presence of PASD positive aggregates was re-analysed in 46 of 55 available specimens of explanted liver from MZ heterozygotes and in 10 of 18 MM homozygotes in whom PASD positive aggregates were found in the first assessment of the explanted liver after LT. PASD positive aggregates were confirmed in all re-analysed specimens, in MZ heterozygotes at significantly higher density and extent (Figure 2). The periseptal distribution of PASD positive aggregates typical for AAT deficiency was present in all the MZ genotype carriers but only in 2 of 10 MM genotype carriers.

**Figure 3:**
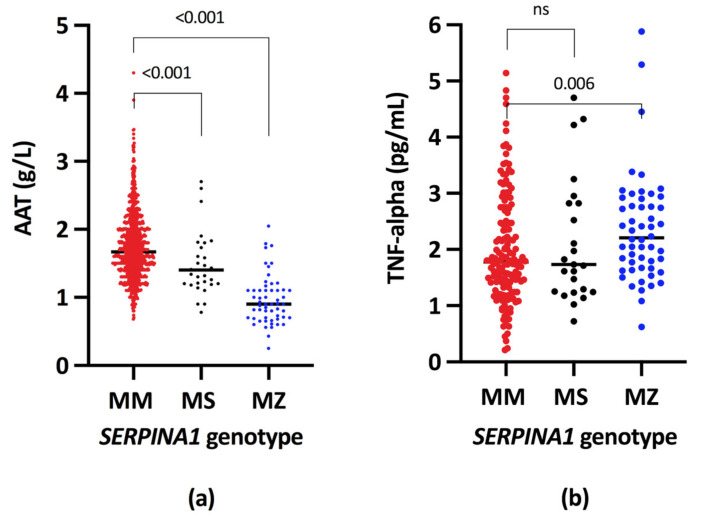
Alpha-1 antitrypsin (**a**) and TNF-alpha levels (**b**) in cirrhotic patients according to the *SERPINA1* genotype. AAT, Alpha-1 antitrypsin; TNF-alpha, Tumour necrosis factor alpha.

Data are presented as medians and ranges. In comparison with MM homozygotes, MZ heterozygotes had approximately 50% levels of serum AAT (MZ: 0.90 g/L, range 0.70–1.1 vs. MM: 1.67; range 1.40–2.00; *p* < 0.001). The lower levels were observed also in MS heterozygotes; however, the difference was less pronounced (MS: 1.40 g/L; range 1.20–1.67, *p* < 0.001) as depicted in Figure 3a. TNF-alpha serum levels were significantly higher only in MZ heterozygotes in comparison with MM homozygotes (MZ: 2.18 pg/mL; range 1.67–2.80 vs. MM: 1.78 pg/mL; range 1.32–2.49; *p* = 0.006). In MS heterozygotes, the serum TNF-alpha levels did not differ from those in MM homozygotes (MS: 1.73 pg/mL; range 1.25–2.82 vs. MM: 1.78 pg/mL; range 1.32–2.49; N.S.), as shown in Figure 3b.

### 2.3. Multivariate Analysis of HCC Risk Factors

**Figure 4:**
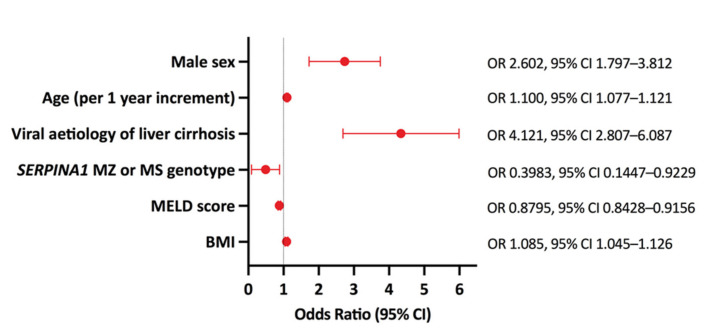
Multivariate logistic regression analysis of risk factor of HCC in the cirrhotic group. BMI, Body Mass Index; CI, Confidence Interval; MELD, Model for End Stage Liver Disease; OR, Odds Ratio.

Multivariate logistic regression analysis of HCC risk factors includes the known factor affecting the risk of HCC and MZ or MS genotype carriage (Figure 4). The MZ or MS genotype carriage maintained its protective effect also in the multivariate analysis. The most powered risk factors were male sex and viral aetiology of liver cirrhosis.

## 3. Discussion

Our results confirmed the postulated hypothesis about the protective effect of the carriage of MZ and MS *SERPINA1* genotypes against HCC in subjects with advanced liver cirrhosis. The findings also documented the previously known fact that MZ genotype carriers are at higher risk of severe liver fibrosis when they suffer from a chronic liver disease [19,20,35,36]. Detailed analysis revealed that, in our cohort of patients with advanced liver cirrhosis of various aetiologies, higher frequency of the MZ genotype was found only in the subgroups of patients with ALD and NASH, whereas carriage of the *SERPINA1* MZ genotype was not a risk factor of liver cirrhosis in chronic liver diseases of viral, autoimmune, cholestatic and metabolic aetiologies. The pro-cirrhotic effect of the MZ genotype was associated with the presence of intrahepatic AAT aggregates, which were of greater extent and density in liver explants in patients with ALD and NASH than in cirrhotic patients with other chronic liver diseases. The pro-cirrhotic effect of the heterozygous carriage of the allele Z was associated with precipitating properties of the AAT variant protein. The S allele, which encodes a non-polymerizing AAT variant protein, was not associated with this effect. Similar results obtained in cohorts of patients with advanced liver cirrhosis were presented by Cacciottolo et al. [20], the authors found that the histological presence of PASD positive aggregates correlated well with the carriage of the Z allele, with a positive predictive value of 95.7%. Interestingly, they initially stratified the patients according to the presence or absence of PASD positive aggregates in the liver. Consistent with our study, they also found a higher prevalence of Z allele carriers in patients with NASH and ALD but not in patients with cholestatic liver disease. In the study by Schaeffer et al. [35] conducted also on the group of patients with advanced liver cirrhosis, the patients were stratified according to the degree of liver dysfunction. The frequency of MZ heterozygotes increased with the severity of liver dysfunction. The impact of heterozygous Z or S allele carriage on the risk of HCC was assessed in none of the studies on patients with advanced liver cirrhosis. In 2019, Strnad et al. [19] in their study of patients with NAFLD and alcohol misusers confirmed the heterozygous carriage of Z allele as an independent factor of liver cirrhosis in both groups. In the ALD cohort, association of liver cirrhosis remained significant also after further adjusting for risk variants of other well-established ALD genetic modifiers (*PNPLA3*, *TM6SF2* and *MBOAT7*). On the other hand, Hakim at al. [37] analysed a large group of patients with chronic liver diseases from the UK Biobank and did not find synergy between Z allele carriage and variants in major genetic modifiers (*PNPLA3*, *TM6SF2* and *HSD17B13*).

In contrast to the pro-cirrhotic effect, our results on the protective effect against HCC of heterozygous Z and S allele carriage seem to be original. The protective role of AAT Z allele against HCC was first studied by Antoury et al. [34]. These authors assessed the role of the Z allele in the allelic model, but the effect of the S allele was not analysed. In our study, we excluded all ZZ homozygotes for the following reasons: firstly, our aim was to evaluate the risk in heterozygous carriers of the Z or S allele; secondly, the number of ZZ homozygotes was low in our cohort, and lastly, the evaluation of the risk of HCC in ZZ homozygotes is complicated by the fact that ZZ homozygotes often develop severe liver fibrosis and cirrhosis at a younger age. However, the recently published data from European Liver Transplantation registry showed that ZZ homozygotes do not represent a group with high risk of HCC; among 90 patients transplanted for AATD, 32 adults and 58 children, only 6 (6.7%) patients developed HCC [38]. Both MZ and MS heterozygotes in our study had low serum AAT levels. Therefore, we speculate that the protective effect against HCC could be attributed to the variant alleles’ carriage, leading to low serum AAT levels.

AAT is generally accepted as a key modulator of TNF-alpha synthesis and signalling [39,40]. Therefore, we focused on TNF-alpha levels. The fact that TNF-alpha level was significantly higher in MZ heterozygotes but not in MS heterozygotes than in MM homozygotes, supports the idea that lower AAT levels may alter various immune mechanisms suppressing hepatocarcinogenesis. It seems unlikely that the increased levels of TNF-alpha in MZ heterozygotes were caused by unrecognized inflammatory lung impairment since none of the MZ heterozygotes suffered from clinically significant lung disease. Furthermore, there was no significant difference in the C-reactive protein serum levels between MM homozygotes and MZ or MS heterozygotes. Indeed, AAT is known to affect the production of other molecules involved in immune signaling, activation of neutrophils and monocytes, and maturation and differentiation of T cells [6,25,41,42,43,44,45,46,47,48]. Alternatively, the anti-apoptotic activity of AAT may also be considered [25,49,50]. Apoptosis is an important mechanism acting against HCC; the anti-HCC drug sorafenib induces apoptosis in HCC and the resistance to this drug is associated with the lack of its apoptotic effect [51]. Similarly, local anaesthetic ropivacaine inhibits HCC proliferation and promotes apoptosis of HCC cells by interaction with caspase-3 [52]. Therefore, we speculate that the low or undetectable level of AAT with the impaired inhibition of caspase-3 could contribute to the anti-HCC effect.

## 4. Conclusions

We conclude that our results support the protective role of *SERPINA1* MZ and MS genotypes against HCC in liver cirrhosis. If validated by others, the finding should prompt studies aiming to decipher the mechanisms behind the protection, which may have exciting consequences for HCC prevention.

## 5. Materials and Methods

### 5.1. Study Design and Eligibility of Patients

The study was designed as a retrospective allelic association study. Cases (cirrhosis group) consisted of 1119 liver transplant candidates with liver cirrhosis of various aetiology referred to the liver transplantation (LT) at our centre between 1 July 1994 and 31 May 2020. Patients referred for LT for primary liver disease other than liver cirrhosis (acute liver failure, polycystic liver disease, vascular liver disease, HCC in non-cirrhotic liver or other liver tumours) were not included in the study. Nine hundred and five patients were enlisted for LT and transplanted for chronic liver failure (CLF), owing to liver cirrhosis using standard criteria for liver dysfunction evaluation (Child–Pugh’s and MELD score) and 214 patients were enlisted for LT for a small HCC in the cirrhotic liver. One hundred and seventy-three patients fulfilled the Milan criteria; the remaining 41 patients complied with San Francisco or up-to-seven criteria, based on pre-transplant imaging techniques. HCC was found incidentally in a further 37 patients by histopathological examination of the explanted liver; 30 of them were inside Milan criteria. Therefore, the final number of patients with HCC in cirrhotic liver was 251. The diagnosis of liver cirrhosis and HCC was confirmed in liver explants, using standard histological staining techniques. Demographic, clinical and laboratory data are shown in the Section 2. All the above-mentioned data were extracted from the electronic hospital information system. The study population was divided into five subgroups, according to the aetiology of liver cirrhosis: alcoholic liver disease (ALD), non-alcoholic steatohepatitis (NASH), autoimmune and cholestatic liver disease (AIH-CHOL), chronic viral hepatitis (VIR) and metabolic liver disease (MET). The assignment of the patients into one of the subgroups was based on their medical history and on the clinical and laboratory data obtained during their pre-transplant work-up. The AIH-CHOL subgroup included patients with autoimmune hepatitis, primary biliary cholangitis, primary sclerosing cholangitis and overlap syndromes. The VIR subgroup included patients with chronic hepatitis B, C or B/D coinfection and the MET subgroup included patients with Wilson’s disease, hereditary haemochromatosis and *SERPINA1* ZZ homozygotes. The *SERPINA1* ZZ homozygotes were included in the study only for the assessment of genotype frequencies among cases and controls. Genotype frequencies of the study group were compared with the genotype frequencies in 3430 healthy subjects (control group), reported in the Czech cross-sectional population study MONICA evaluating cardiovascular disease and hypertension risk in the Czech population [53]. All the study subjects as well as all the individuals of the control group were of Caucasian origin.

### 5.2. Genotyping

Patients’ DNA was isolated from the peripheral blood using the Qiagen QIAamp DNA blood mini kit (Qiagen, Hilden, Germany). The rs28929474 locus (PI*Z) in *SERPINA1* was analysed using the TaqMan SNP assay No. C_34508510_10 (Thermo Fisher Scientific, Waltham, MA). The rs17580 locus (PI*S) in *SERPINA1* was genotyped with the TaqMan SNP assay No. C_594695_20. Genotyping was performed according to the manufacturer’s protocol. Both assays were run on the Applied Biosystems ABI 7300 Real-Time PCR instrument (Thermo Fischer Scientific) and Applied Biosystem QuantStudio6 Flex Real-Time PCR System.

### 5.3. AAT and TNF-Alpha Serum Levels Assessment

Serum AAT concentration was measured in all subjects during the pre-transplant work-up by the immunoturbidimetric method, with expected normal range 0.88–1.74 g/L.

Serum levels of TNF-alpha were determined in blood samples collected immediately before liver transplantation. At the time of blood sampling, the patients had no symptoms or laboratory signs of infection. The samples were frozen immediately after serum separation and stored at −80 °C. TNF-alpha was assessed in 53 serum samples of *SERPINA1* MZ heterozygotes, in 25 MS heterozygotes and in 156 sex-, age- and liver cirrhosis aetiology matched group of *SERPINA1* MM homozygotes.

Quantitative determination of TNF-alpha was performed with the Quantikine HS ELISA human TNF-alpha immunoassay (R&D Systems, Abingdon, UK). All standards, controls and samples were analysed in duplicates, and the duplicate readings were averaged. None of the duplicates showed coefficient variability (CV) higher than 50%; therefore, all samples were included in further analysis.

### 5.4. Explanted Liver Tissue Histological Analysis

The stored histopathological samples from explanted livers were evaluated, and one representative paraffin block from left and right liver lobe was selected and processed for histopathological examination. Selected stains included haematoxylin and eosin (HE), and periodic acid-Schiff diastase (PASD). HE was performed on sections of 3 µm and PASD on 4 µm thickness. The PASD positive intrahepatic inclusions were assessed by semiquantitative grading system including their density, extent and distribution. The evaluation of the density was based on the previous study of Clark et al. [16], using a three-tier scoring system, in which a number of positive hepatocytes was counted per one high power field of microscope and graded as 0—none; 1—rare, < 5 hepatocytes with globules; 2—few, 5–20 hepatocytes with globules; and 3—numerous, ≥ 20 hepatocytes with globules. The extent was characterized by a portion of affected liver tissue in the histological slide and was graded as 0—no; 1—scarce; 2—focal; and 3—diffuse. Furthermore, the presence of characteristic periseptal distribution of the globules at the periphery of cirrhotic nodules was included as well.

### 5.5. Statistical Analysis

Continuous variables are presented as means and standard deviations, whereas categorical variables are expressed as frequencies (%). Categorical data were analysed, using the chi-square test. For continuous data, Student’s *t*-test and one-way ANOVA or the non-parametric Mann–Whitney and Kruskall–Wallis tests were used appropriately. Testing for genetic associations was performed as described in [54]. Risk factors were examined, using multivariate logistic regression analysis. All statistical analyses were two-sided and *p* value of <0.05 was considered statistically significant throughout the study. Statistical analysis was performed, using GraphPad Prism v8.2.1 for Mac, GraphPad Software, San Diego, CA, USA (www.graphpad.com) and R programming language v3.2.0 (www.r-project.org).

### 5.6. Ethics Statement

This study was approved by the Ethics Committee of the Thomayer’s Hospital and Institute for Clinical and Experimental Medicine, Prague, Czech Republic, and was carried out in compliance with the Helsinki Declaration. All study participants gave written consent to the storage of blood samples and agreed to using blood for future research including genetic testing. The written consent was obtained before enlistment for liver transplantation.

## Figures and Tables

**Table 1:** *SERPINA1* genotype frequency in cases and in controls.

Genotype	Cases (Cirrhosis Group) (*n* = 1119)	Control Group (MONICA Study) (*n* = 3430)	*p* Value
MM	1021	3240	N.A.
MZ	55	87	<0.0001 OR 1.986; 95% CI 1.413–2.806
MS	32	101	N.S.
SZ	1	0	N.A.
ZZ	10	0	<0.0001 OR Infinity; 95% CI 9.316–Infinity
SS	0	2	N.A.

**Table 2:** *SERPINA1* genotype distribution according to the aetiology of liver cirrhosis in comparison with the control group.

*SERPINA1* Genotype	Controls (*n* = 3430)	ALD Cirrhosis Subgroup (*n* = 360)	*p* Value	NASH Cirrhosis Subgroup (*n* = 146)	*p* Value	Pooled Subgroup of Other Cases * (*n* = 603)	*p* Value
MM	3240	327	N.A.	125	N.A.	569	N.A.
MZ	87	24	<0.001 OR 2.745 95% CI 1.744–4.325	16	<0.001 OR 4.729 95% CI 2.733–8.298	15	N.S.
MS	101	9	N.S.	4	N.S.	19	N.S.
SZ	0	0	N.A.	0	N.A.	0	N.A.
ZZ	0	0	N.A.	0	N.A.	0	N.A.
SS	2	0	N.A.	0	N.A.	0	N.A.

* Pooled subgroup of other cases included viral aetiology cirrhosis subgroup, autoimmune and cholestatic cirrhosis subgroup, and metabolic aetiology cirrhosis subgroup; ALD, alcoholic liver disease; CI, confidence interval; NASH, non-alcoholic steatohepatitis; OR, Odds ratio.

**Table 3:** Aetiology of liver cirrhosis and occurrence of HCC in liver cirrhosis according to the *SERPINA1* genotype.

		MM (%) (*n* = 1021)	MZ (%) (*n* = 55)	*p* Value MM vs. MZ	MS (%) (*n* = 32)	*p* Value MM vs. MS
Aetiology of liver cirrhosis	ALD	327 (32%)	24 (43.6%)	0.002	9 (28.1%)	N.S.
	NASH	125 (12.2%)	16 (29.1%)		4 (12.5%)	
	VIR	209 (20.5%)	8 (14.6%)		3 (9.4%)	
	AIH-CHOL	302 (29.6%)	6 (10.9%)		12 (37.5%)	
	MET	58 (5.7%)	1 (1.8%)		4 (12.5%)	
HCC in liver cirrhosis		243 (23.8%)	5 (9.1%)	0.0085 *	1 (0.4%)	0.0044 **

* OR 0.3202; 95% CI 0.1361–0.7719; ** OR 0.1522; 95% CI 0.02941–0.7882; AIH-CHOL, autoimmune and cholestatic liver disease; ALD, alcoholic liver disease; CI, confidence interval; HCC, hepatocellular carcinoma; MET, metabolic liver disease; NASH, non-alcoholic steatohepatitis; OR, odds ratio; VIR, chronic viral hepatitis.

**Table 4:** Demographic and laboratory characteristics of patients grouped according to *SERPINA1* genotypes.

	MM (*n* = 1021)		MZ (*n* = 55)			MS (*n* = 32)		
	Number of Analysed Subjects	Parameter	Number of Analysed Subjects	Parameter	*p* Value (MZ vs. MM)	Number of Analysed Subjects	Parameter	*p* Value (MS vs. MM)
Age, years	1021	56.7 (46.9–63.3)	55	54.2 (48.4–62.2)	0.279	32	48.2 (41.7–55.8)	0.004
BMI, kg/m^2^	1021	25.5 (22.4–28.7)	55	27.5 (24.9–30.0)	0.004	32	26.2 (21.3–29.7)	0.894
MELD score	1008	14 (11–17)	55	17 (14–20)	<0.001	32	17 (15–19)	0.001
Serum A1AT, g/L	983	1.67 (1.40–2.00)	55	0.90 (0.70–1.10)	<0.001	32	1.40 (1.20–1.67)	<0.001
Serum TNFA, pg/L	156	1.78 (1.32–2.49)	53	2.18 (1.67–2.80)	0.006	25	1.73 (1.25–2.82)	0.742
Serum CRP, mg/L	689	8.3 (0–66.0)	45	8.0 (0.4–69.4)	0.740	23	9.0 (0.6–76.4)	0.640
Serum Creatinine, μmol/L	1014	74 (61–90)	55	78 (67–104)	0.025	32	69 (61–86)	0.632
Serum Bilirubin, μmol/L	1014	43 (25–86)	55	63 (41–88)	0.023	32	70 (35–92)	0.032
Prothrombin time, INR	1009	1.30 (1.20–1.50)	55	1,49 (1.33–1.70)	<0.001	32	1.49 (1.30–1.70)	0.005
Serum Albumin, g/L	1014	29.5 (25.2–34.0)	55	23.7 (21.6–27.9)	<0.001	32	30.0 (25.5–33.6)	0.931
Intrahepatic A1AT aggregates, N	953	18	55	52	<0.0001	29	1	0.4372

## Data Availability

Data supporting reported results are available upon request at the authors.
